# Treatment of isoniazid-resistant pulmonary tuberculosis

**DOI:** 10.1186/1471-2334-8-6

**Published:** 2008-01-23

**Authors:** Yee Hyung Kim, Gee Young Suh, Man Pyo Chung, Hojoong Kim, O Jung Kwon, Seong Yong Lim, Si Young Lim, Won-Jung Koh

**Affiliations:** 1 Division of Pulmonary and Critical Care Medicine, Department of Medicine, Samsung Medical Center, Sungkyunkwan University School of Medicine, Seoul, Korea; 2 Division of Pulmonary and Critical Care Medicine, Department of Internal Medicine, Kangbuk Samsung Hospital, Sungkyunkwan University School of Medicine, Seoul, Korea

## Abstract

**Background:**

Although resistance to isoniazid (INH) is the most common form of drug resistance seen among *Mycobacterium tuberculosis *isolates, there have been few studies on the efficacy and optimal duration of treatment for patients with INH-resistant tuberculosis (TB).

**Methods:**

We evaluated retrospectively the treatment outcomes of 39 patients who were treated for INH-resistant pulmonary TB. The treatment regimens consisted of a 12-month regimen of rifampin (RIF) and ethambutol (EMB), with pyrazinamide (PZA) given during the first 2 months (2HREZ/10RE) (*n *= 21), a 9-month regimen of RIF and EMB with PZA during the first 2 months (2HREZ/7RE) (*n *= 5), and a 6-month regimen of RIF, EMB, and PZA (2HREZ/4REZ) (*n *= 13). After drug susceptibility testing confirmed the INH-resistance of the isolated *M. tuberculosis *strains, INH was discontinued for all the patients.

**Results:**

Among the 39 patients, treatment was successfully completed by 36 patients (92%). However, treatment failure occurred, and acquired resistance to other first-line drugs, such as RIF, developed in three patients (8%). Cavitary and bilateral extensive lesions were commonly found in the chest radiographs of the patients who exhibited treatment failure.

**Conclusion:**

These findings underline the seriousness of concerns regarding treatment failure and the development of multidrug-resistant TB in patients with INH-resistant TB following treatment with recommended regimens.

## Background

Recent global surveys have revealed that drug-resistant tuberculosis (TB) exists in virtually every location examined [1-3]. Among drug-resistant *M. tuberculosis *isolates, resistance to isoniazid (INH) was the most commonly observed type [1].

Previous studies have suggested that standard, 6-month, four-drug regimens may be effective in the treatment of INH-resistant TB [4]. However, the guidelines for the treatment of TB published recently by the American Thoracic Society and British Thoracic Society have stated that it would be more prudent either to administer pyrazinamide (PZA) continuously throughout the 6-month treatment period or to prolong the duration of treatment [5-7].

It is very surprising that the efficacies of these recommended regimens have not been evaluated fully. Recently, we encountered a case of pulmonary TB with acquired multidrug resistance (MDR) during a 12-month treatment of INH-resistant TB with RIF and EMB, with PZA administered during the first 2 months [8]. The aims of the present study were to evaluate retrospectively the treatment outcomes for INH-resistant TB with the various regimens recommended in the previous guidelines and published literature.

## Methods

We searched the database of all notified cases of pulmonary TB diagnosed and treated in our institutions between July 2001 and June 2005. Permission was obtained from the Institutional Review Board to review and publish retrospectively the patients' records.

The drug susceptibilities of the *M. tuberculosis *isolates were determined by the absolute concentration method, using Lowenstein-Jensen medium at the Korean Institute of Tuberculosis [9]. The drugs and their critical concentrations for resistance were as follows: INH, 0.2 μg·mL^-1^; RIF, 40 μg·mL^-1^; SM, 4 μg·mL^-1^; and EMB, 2 μg·mL^-1^. PZA susceptibility was determined by the pyrazinamidase test [10].

INH resistance was defined as resistance to INH alone or INH plus streptomycin (SM). In total, 53 patients with INH-resistant pulmonary TB were identified and their medical records were analyzed retrospectively. We excluded patients who were lost to follow-up (*n *= 6) and those who had been transferred to other institutions, usually to their referring institutions before completion of treatment (*n *= 7). We also excluded one patient who died of gastrointestinal bleeding during treatment for TB.

A patient was designated as being cured if conversion from positive to negative sputum culture was achieved after the start of treatment and the patient remained culture-negative throughout the period of treatment. Sputum culture conversion was defined as the time in months from the time treatment was started to the time at which the first negative sputum culture was obtained. Treatment failure was defined in bacteriologic terms for patients who, while on treatment, continued to be, or reverted to being, smear-positive and/or culture-positive 5 months or more after commencing treatment [11,12]. "Treatment completed" was the definition given to those patients who had completed treatment but who did not meet the criteria to be classified as instances of cure or failure. In this study, both cure and treatment completed were regarded as treatment successes.

## Results

### Baseline characteristics

The baseline clinical characteristics of the 39 patients with INH-resistant pulmonary TB are listed in Table 1.

**Table 1:** Baseline characteristics of 39 patients with isoniazid-resistant pulmonary tuberculosis

Patient characteristics		Treatment Success (n = 36)	Treatment failure (n = 3)
Sex	Male	24 (67%)	2 (67%)
	Female	12 (33%)	1 (33%)
Median age in years (range)		41 (24–71)	55 (55–72)
Comorbidity	Diabetes mellitus	2	2
	Malignancy	4	0
	Chronic liver disease	2	1
	Chronic pulmonary disease	2	1
	Others	5	0
Smear-positive		20 (56%)	3 (100%)
Type of resistance	Isoniazid	31 (86%)	3 (100%)
	Isoniazid and streptomycin	5 (14%)	0
History of treatment	New	23 (64%)	2 (67%)
	Relapse	10 (28%)	1 (33%)
	Treatment after default	3 (8%)	0
Radiographic findings	Cavitary lesion	13 (36%)	2 (67%)
	Bilateral lesion	15 (42%)	2 (67%)
	Extensive lesion	6 (17%)	2 (67%)

Twenty-three (59%) patients were smear-positive and culture-positive, and 16 (41%) patients were smear-negative and culture-positive. Cavitary lesions were identified in the chest radiographs of 15 (38%) patients. Bilateral lesions were found in 17 (44%) patients, and extensive lesions, which were defined as the involvement of two or more lobes in the same lung, were detected in 8 (21%) patients.

### Treatment regimens

All of the patients initially received daily therapy that comprised INH, RIF, EMB, and PZA, which was strongly recommended by the National Tuberculosis Program [13-15]. After drug susceptibility testing confirmed the INH-resistance of the isolated *M. tuberculosis *strains, INH treatment was discontinued for all the patients. For 13 (33%) patients in whom INH-resistance was identified before the end of the 2-month initial phase of treatment, PZA was included in the treatment regimen for the entire 6 months (2HREZ/4REZ). In 25 (64%) patients, drug susceptibility test results were available during the continuation phase of treatment. In these 25 patients, RIF and EMB were continued for 12 months in 21 (54%) patients (2HREZ/10RE) and for 9 months in 5 (13%) patients (2HREZ/7RE). All of the regimens were administered daily for the full duration of therapy.

Treatment compliance was assessed by comparing the number of administered treatment doses to the number of treatment doses scheduled each month. There were no consistently missing doses corresponding to ≥ 10 days of monthly scheduled doses of medication for all the patients. Trained nurses checked the adherence of patients and called patients who did not show up for their monthly appointment. All the patients completed the treatment regimen without modification.

### Treatment outcomes

Treatment was successfully completed in 36 patients (92%), and 35 (90%) were cured. Table 2 shows the elapsed times for sputum culture conversion for these patients with INH-resistant pulmonary TB. The majority (30/36, 83%) of the patients converted from culture-positive to culture-negative status after 1 month of treatment.

**Table 2:** Treatment outcome and time from start of treatment to conversion of sputum culture among 39 patients with isoniazid-resistant pulmonary tuberculosis

Treatment regimen	Number of patients	Treatment success				Treatment failure
			
		Sputum conversion from positive to negative culture at				
			
		1 month	2 months	3 months	Follow-up sputum sample not available*	
2HREZ/10RE	Smear-positive (n = 12)	7	1	1	1	2
	Smear-negative (n = 9)	8	1	-	-	-
2HREZ/4REZ	Smear-positive (n = 9)	6	1	1	-	1
	Smear-negative (n = 4)	4	-	-	-	-
2HREZ/7RE	Smear-positive (n = 2)	2	-	-	-	-
	Smear-negative (n = 3)	3	-	-	-	-

Total (n = 39)		30	3	2	1	3

* One patient, who was diagnosed as having culture-positive pulmonary tuberculosis based on bronchoscopic examination, could not expectorate sputum after the initiation of treatment. This patient could not expectorate sputum after the initiation of treatment. The radiographic abnormality disappeared completely after the treatment, and this patient was classified as a treatment success.

2HREZ/10RE: 12-month regimen of rifampin (RIF) and ethambutol (EMB), with pyrazinamide (PZA) during the first 2 months; 2HREZ/4REZ: 6-month regimen of RIF, EMB, and PZA; 2HREZ/7RE: 9-month regimen of RIF and EMB, with PZA during the first 2 months. After drug susceptibility testing confirmed the INH-resistance of the isolated *M. tuberculosis *strains, INH treatment was discontinued for all the patients.

However, treatment failure occurred in three patients (8%). In two patients, progression to resistance to INH and RIF (MDR) was identified during the treatment of 2HREZ/10RE regimen. Sputum conversion of smears and cultures was initially achieved after 3 and 4 months of treatment in these patients. However, the sputum culture returned to positivity and the isolated organism was found to be resistant to INH and RIF after 9 and 10 months of treatment. In one patient, sputum smears and cultures remained persistently positive after 5 months of treatment of 2HREZ/4REZ and resistance to both INH and PZA developed. Initial sputum AFB smear was positive in all these patients with treatment failure. Two patients had cavitary lesion and two patients had bilateral lesion on their initial chest radiographs.

## Discussion

A review of 12 controlled trials conducted in Africa, Hong Kong, and Singapore had revealed that the standard 6-month, four-drug regimens may be effective in the treatment of INH-resistant TB [4]. However, that review [4] also stated that, "the proportion of relapse in regimens containing RIF throughout was approximately twice as high in patients with initial resistance to INH as in those with sensitive strains." In addition, one large-scale multinational cohort study revealed that treatment failure is higher among patients with any INH resistance other than MDR in both new cases and retreatment cases who have received short-course chemotherapy with INH, RIF, PZA, and either EMB or SM [16]. Another study also found that INH resistance was strongly associated with treatment failure during standardized short-course chemotherapy for TB, and many patients involving treatment failures acquired new MDR [17].

The American Thoracic Society and British Thoracic Society have recommended several treatment regimens for the treatment of INH-resistant TB, which included 6REZ, 2REZ/10RE, and 12RE [5-7]. In addition, some studies have suggested that a 9-month regimen of 2REZ(S)/7RE may be valuable for the treatment of INH-resistant TB [18,19]. However, in spite of the presence of the different treatment regimens listed above, the optimal treatment regimen and duration remain unclear, as the efficacies of these regimens have not been fully evaluated in a prospective randomized fashion. Indeed, many physicians treat INH-resistant TB patients with widely varying regimens in daily clinical practice [20].

In the present study, the treatment success rate was 92% and treatment failure occurred in three cases (8%). The overall success rates of 90% (19/21) for 2HREZ/10RE, 92% (12/13) for 2HREZ/4REZ, and 100% (5/5) for 2HREZ/7RE. One case of treatment failure developed during treatment with the 2HREZ/4REZ regimen and two cases of treatment failure developed during treatment with 2HREZ/10RE. Importantly, the acquisition of additional resistance to other first-line drugs occurred in each of these cases. The cavitary and bilateral extensive lesions seen in the chest radiographs of these patients were commonly associated with treatment failure, and the sputum smears of these patients were positive.

Interestingly, the recently revised treatment guidelines by the American Thoracic Society and the World Health Organization state that fluoroquinolones may augment the 6REZ regimen for the treatment of patients with INH-resistant pulmonary TB who have more extensive disease [6,21]. During the study period, however, the flouroquinolones were not used in the treatment of INH-resistant pulmonary TB in our institutions.

The present study has many important limitations that are generally inherent to retrospective analyses. The effectiveness of various regimens for the treatment of INH-resistant TB cannot be deduced from our study, since the numbers of patients who received the various regimens are insufficient for the purpose of comparison. More importantly, the assignment of the treatment regimens was not random. In addition, our institutions did not perform directly observed therapy (DOT). Then, the possibility of poor compliance of treatment regimen could not be completely excluded.

## Conclusion

Our report underlines the seriousness of concerns regarding treatment failure and the development of MDR-TB in patients with INH-resistant TB following treatment with recommended regimens.

## Competing interests

The author(s) declare that they have no competing interests.

## Authors' contributions

YHK, WJK, OJK, and SYL designed the study. YHK and SYL collected and analyzed data. YHK and WJK primarily wrote the manuscript. GYS, MPC, and HK provided valuable insight for revising the manuscript. All authors read and approved the final manuscript.

## Pre-publication history

The pre-publication history for this paper can be accessed here:


